# Systemic Hepatic-Damage Index for Predicting the Prognosis of Hepatocellular Carcinoma after Curative Resection

**DOI:** 10.3389/fphys.2017.00480

**Published:** 2017-07-18

**Authors:** Xing-hui Gao, Shuang-shuang Zhang, Hao Chen, Yu-Hui Wang, Chun-Hui Yuan, Fu-Bing Wang

**Affiliations:** ^1^Department of Laboratory Medicine, Zhongnan Hospital of Wuhan University Wuhan, China; ^2^Department of Dermatology, Shanghai Dermatology Hospital, Tongji University Shanghai, China; ^3^Department of Pathology, Zhongnan Hospital of Wuhan University Wuhan, China

**Keywords:** system hepatic-damage index, hepatocellular carcinoma, recurrence, prognosis, survival

## Abstract

**Purpose:** We have developed a systemic hepatic-damage index (SHI) based on serum total cholesterol (TC) and high density lipoprotein levels (HDL) and determined its prognostic significance in hepatocellular carcinoma (HCC) patients undergoing resection.

**Experimental Design:** The SHI was analyzed in the training cohort of 188 HCC patients and in the validation cohort of 177 HCC patients. The systemic immune-inflammation index (SII) scores in the validation cohorts were also measured. Area under the receiver operating characteristic curve (AUC) was used to explore the prediction accuracy in HCC patients.

**Results:** An optimal cutoff point for the SHI of 2.84 stratified the HCC patients into high SHI (>2.84) and low SHI (≤2.84) groups in the training cohort. Univariate and multivariate analyses revealed the SHI was an independent predictor for overall survival and relapse-free survival, and prognostic for patients with negative α-fetoprotein and Barcelona Clinic Liver Cancer stage 0+A. The AUCs of the SHI for survival and recurrence were higher than other conventional clinical indices. Low SHI significantly correlated with vascular invasion. The SII scores were significantly higher in patients with low SHI compared with those with high SHI. HCC patients in SHI ≤ 2.84 group had shorter recurrence time and lower survival rate than HCC patients in SHI > 2.84 group.

**Conclusions:** The SHI was a potential biomarker for assessing HCC prognosis, and improving SHI level in HCC patients may be a promising therapeutic strategy decision. The poor outcome in HCC patients with low SHI scores might increase SII scores, increasing the possibility of recurrence and metastasis.

## Introduction

Hepatocellular carcinoma (HCC) is the most common malignancy with an increasing mortality rate worldwide. So far, surgical resection remains the most effective treatment for HCC patients; however, the prognosis of HCC patients who underwent curative resection remains unsatisfactory because of high recurrence possibility and metastasis incidence (Ji et al., 2009; Forner et al., 2012; Torre et al., 2015). The utility of conventional clinicopathological parameters, including histopathological characteristics, is insufficient for identifying patient subpopulations at high risk of tumor recurrence and metastasis. Therefore, it is urgent to develop new biomarkers for identifying tumor recurrence and metastasis so that post-operative rational adjuvant treatments can be provided timely.

The reasons for the high recurrence/metastasis rate in HCC are complex and multifactorial (Forner and Raoul, 2011). Hepatic damage is an important cause of recurrence and metastasis in HCC. In addition to tumor cells, immune and inflammatory cells such as neutrophils, platelets, and lymphocytes also contribute to tumor cell invasion into the peripheral blood, where tumor cells can survive and reseed distant organs (Chaffer and Weinberg, 2011; Hanahan and Weinberg, 2011; Casadei et al., 2016).

HBV infection leads to liver damage, relating to changes of the lipid metabolism. Several studies have shown that lower serum total cholesterol (TC) level correlated with cancer recurrence in HCC patients with hepatic resection, revealing the possibility to predict surgical outcome by preoperative clinical physiological parameters. Poorer surgical outcome in HCC patients with lower preoperative serum TC level might be caused by long-term liver dysfunction and malnutrition, implying the potential role of lipid composition in determination of operative outcomes in HCC patients (Irshad et al., 2007; Wang et al., 2014; Lee et al., 2016; Zhang et al., 2017). Recent studies have reported that serum high-density lipoprotein (HDL) is in relation to incident cancers especially breast cancer. These findings imply that HDL might play a major role in tumorigenesis and tumor progression (Lim et al., 2007; Li et al., 2016; Wang et al., 2016). However, an integrated indicator based on serum TC and HDL levels, which might be superior to reflect the correlation between hepatic damage and tumor stages, has not yet been reported in HCC. Moreover, the potential effects of serum TC and HDL levels on HCC recurrence and metastasis have not been elaborated.

In this study, an index based on serum TC and HDL levels, designated as the systemic hepatic-damage index (SHI), has been developed. The prognostic value of the SHI in HCC patients who underwent surgery was evaluated in two independent cohorts. The correlation between the SHI and systemic immune-inflammation index (SII) scores was also explored. We found that the SHI was a potential biomarker for predicting prognosis of HCC patients undergoing resection and that high recurrence rate in patients with low SHI score might be due to increased SII scores from the host.

## Materials and methods

### Study design

Ethical approval for the use of human subjects was obtained from the Research Ethics Committee of Zhongnan Hospital. From January 2011 to June 2013, we recruited 188 HBV-HCC patients undergoing resection at Zhongnan Hospital as a training cohort. We next recruited a validation cohort of 177 HBV-HCC patients who underwent resection from July 2013 to December 2015 (Table 1). HCC was defined according to the data of imaging scans and biochemical assays, and diagnosis was confirmed by histopathology according to the criteria of the American Association for the Study of Liver Diseases guidelines. The stage of HCC was determined according to the BCLC guidelines (Bruix and Sherman, 2011). Tumor size was defined using the diameter of the single largest lesion. Tumor differentiation was defined using the Edmondson grading system (Wittekind, 2006).

**Table 1:** The clinicopathologic characteristics of patients in the training and validation cohorts.

**Characteristics**		**Training cohort**		**Validation cohort**		***P***
		***N* = 188**	**%**	***N* = 177**	**%**	
Age (years)	≤50	69	36.70	68	38.42	0.747
	>50	119	63.30	109	61.58	
Sex	Women	26	13.83	32	18.08	0.316
	Men	162	86.17	145	81.92	
HBsAg	Negative	22	11.70	21	11.86	1.000
	Positive	166	88.30	156	88.14	
Liver cirrhosis	No	40	21.28	43	24.29	0.533
	Yes	148	78.72	134	75.71	
No. of tumors	Single	151	80.32	148	83.62	0.497
	Multiple	37	19.68	29	16.38	
Tumor size, cm	≤5	119	63.30	107	60.45	0.591
	>5	69	36.70	70	39.55	
Tumor encapsulation	Complete	114	60.64	121	68.36	0.128
	None	74	39.36	56	31.64	
Satellite lesion	No	165	87.77	161	90.96	0.397
	Yes	23	12.23	16	9.04	
Vascular invasion	No	118	62.77	104	58.76	0.454
	Yes	70	37.23	73	41.24	
Tumor differentiation	I–II	116	61.70	114	64.41	0.664
	III–IV	72	38.30	63	35.59	
Child-Pugh score	A	177	94.15	159	90.87	0.175
	B	11	5.85	18	9.13	
BCLC stage	0+A	137	72.87	139	78.53	0.224
	B+C	51	27.13	38	21.47	
AFP, ng/mL		2,391 ± 588		3,328 ± 858		0.360
ALT, U/L		36.27 ± 2.08		38.86 ± 2.01		0.415
TB, umol/L		12.40 ± 0.40		11.80 ± 0.36		0.270
Albumin, g/L		40.24 ± 0.23		40.40 ± 0.26		0.650
PT, s		12.38 ± 0.07		12.46 ± 0.08		0.458
TC, mmol/L		4.10 ± 0.06		4.19 ± 0.07		0.317
HDL, mmol/L		1.25 ± 0.02		1.27 ± 0.03		0.718
SHI		5.23 ± 0.14		5.35 ± 0.16		0.560

*AFP, α-fetoprotein; ALT, alanine aminotransferase; HBsAg, hepatitis B surface antigen; BCLC, Barcelona Clinic Liver Cancer; TB, total bilirubin; PT, prothrombin time;TC, total cholesterol; HDL, high-density lipoprotein; SHI, systemic hepatic-damage index*.

### Follow-up and tumor recurrence

Follow-up for HCC patients ended in July 2016. The time to recurrence (TTR) was defined as the interval between surgery and any diagnosed intrahepatic or extrahepatic recurrence. The overall survival (OS) was defined as the interval between the date of surgery and the date of death, or the interval between surgery and the last observation.

### Systemic hepatic-damage index

The serum TC and HDL concentrations were determined by immunoturbidimetry, using Olympus AU5831 automated biochemistry analyzer. Serum samples were collected 2 days before surgery, and stored at −80°C before detection. The SHI was defined as follows: SHI = TC × HDL. The cutoff value of SHI for predicting tumor recurrence in the training cohort was determined by the X-tile 3.6.1 software (Camp et al., 2004). Results from X-Tile analysis revealed an optimal cutoff point for the SHI 2.84 in the training cohort (Supplementary Figure 1). Thus, patients were stratified into SHI high (>2.84) or low (≤2.84) groups for all subsequent analyses.

### Systemic immune-inflammation index

The SII was defined as follows: SII = P×N/L, where P, N, and L were the preoperative peripheral platelet, neutrophil, and lymphocyte counts, respectively (Hu et al., 2014).

### Statistical analysis

Statistical analyses were performed using SPSS 16.0 software. Experimental values for continuous variables were expressed as the mean ± standard error of the mean. The chi-squared test, Fisher's exact probability tests and the Student's *t*-test were used to evaluate the significance of differences in data between groups. If variances within groups were not homogeneous, the non-parametric Mann–Whitney test or the Wilcoxon signed-rank test was used. Kaplan–Meier survival curves and log-rank tests was used to analyze the relationships between serum SHI scores and TTR and OS, and *p* < 0.05 was considered statistically significant.

## Results

### Patient characteristics

The clinicopathologic characteristics of HBV-HCC patients are showed in Table 1. In the training cohort, 89 of 188 HBV-HCC patients undergoing resection suffered recurrence with a median follow-up time of 30.9 months (range 0.3–36.8 months) and 51 of 188 HBV-HCC patients died before the last follow-up (median follow-up 33.0 months, range 3.2–36.8 months). The validation cohort comprised 61 of 177 HBV-HCC patients who confirmed recurrence with a median follow-up time of 20.0 months (range 1.0–30.3 months), and 140 HBV-HCC patients were still alive at a median follow-up of 22.5 months (range 4.0–30.3 months). In the training and validation cohorts, all clinicopathathologic characteristics were similar (Table 1).

### Prognostic value of SHI in the training cohort

**Figure 1:**
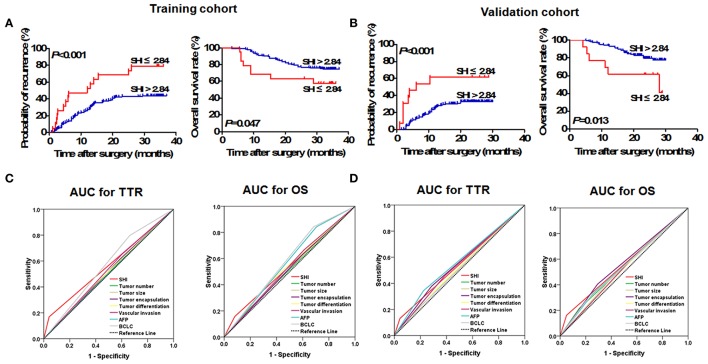
Prognostic significance of SHI in HCC patients underwent curative resection. Prognostic significance of SHI in HCC patients undergoing resection. The Kaplan–Meier analysis of OS and TTR for SHI in the training **(A)** and validation **(B)** cohorts. Predictive ability of SHI was compared with other clinical parameters by ROC curves in the training **(C)** and validation **(D)** cohorts.

Using the X-tile 3.6.1 software, the optimal cutoff value (2.84) was set as to stratify HBV-HCC patients into low SHI (≤2.84) and high (>2.84) groups (Supplementary Figure 1). Kaplan-Meier analysis showed that TTR of HBV-HCC patients with low SHI was significantly shorter (*p* < 0.001, Figure 1A) and had higher recurrence rates than those with a high SHI (78.95 vs. 43.79%). Furthermore, the OS of HBV-HCC patients with low SHI was significantly shorter (*p* = 0.047, Figure 1A) than those with high SHI (42.11 vs. 25.44%). In multivariate analysis, the SHI was an independent indicator for TTR [Hazard ratio (HR), 2.97; 95% confidence interval (CI): 1.58–5.58; *p* = 0.001; Table 2] and OS (HR, 2.82; 95% CI: 1.25–6.39; *p* = 0.013; Table 2) of HBV-HCC patients. The area under curve (AUC) was used to assess the discrimination ability of SHI and clinical factors for TTR and OS (Figure 1B). The AUC for SHI was 0.56 (95% CI, 0.48–0.65; Sensitivity, 0.169; Specificity, 0.959; Youden's index, 0.128) for TTR, and 0.54 (95% CI, 0.44–0.663; Sensitivity, 0.157; Specificity, 0.919; Youden's index, 0.076) for OS (Supplementary Table 1), which was the strongest factor among clinical factors [tumor size, tumor number, tumor differentiation, tumor encapsulation, vascular invasion, α-fetoprotein (AFP), and Barcelona Clinic Liver Cancer (BCLC) stage] for predicting tumor recurrence and survival of HBV-HCC patients.

**Table 2:** Multivariate Cox regression analyses in the training and validation cohort.

**Variables**	**TTR**		**OS**	
	**HR (95% CI)**	***P***	**HR (95% CI)**	***P***
**TRAINING COHORT**				
AFP, ng/ml (>400 vs. ≤400)	0.832 (0.488–1.416)	0.497	0.390 (0.171–0.892)	**0.026**
No. of tumors, (multiple vs. single)	0.769 (0.414–1.425)	0.403	0.979 (0.434–2.205)	0.958
Tumor size, cm (>5 vs. ≤5)	0.781 (0.461–1.326)	0.361	1.115 (0.564–2.204)	0.754
Tumor encapsulation, (none vs. complete)	0.893 (0.568–1.404)	0.623	1.089 (0.597–1.984)	0.781
Satellite lesion, (yes vs. no)	1.167 (0.569–2.395)	0.673	1.754 (0.692–4.444)	0.236
Vascular invasion, (yes vs. no)	0.987 (0.598–1.631)	0.961	0.864 (0.448–1.665)	0.662
Tumor differentiation, (III-IV vs. I-II)	1.072 (0.673–1.706)	0.771	1.416 (0.770–2.605)	0.264
Child-Pugh score, (A vs. B)	1.777 (0.783–4.033)	0.169	2.248 (0.848–5.961)	0.103
BCLC stage, (0+A vs. B+C)	0.593 (0.323–1.090)	0.092	0.354 (0.147–0.853)	**0.021**
SHI (>2.84 vs. ≤2.84)	2.969 (1.579–5.581)	**0.001**	2.824 (1.248–6.388)	**0.013**
**VALIDATION COHORT**				
AFP, ng/ml (>400 vs. ≤400)	1.610 (0.916–2.831)	0.098	1.429 (0.683–2.989)	0.343
No. of tumors, (multiple vs. single)	0.883 (0.415–1.875)	0.745	0.820 (0.308–2.186)	0.692
Tumor size, cm (>5 vs. ≤5)	0.951 (0.473–1.915)	0.889	0.873 (0.354–2.154)	0.768
Tumor encapsulation, (none vs. complete)	0.650 (0.373–1.132)	0.128	2.264 (1.087–4.718)	**0.029**
Satellite lesion, (yes vs. no)	1.244 (0.551–2.828)	0.599	1.348 (0.478–3.802)	0.572
Vascular invasion, (yes vs. no)	0.800 (0.434–1.475)	0.475	0.469 (0.206–1.069)	0.072
Tumor differentiation, (III–IV vs. I–II)	1.051 (0.605–1.825)	0.860	0.812 (0.388–1.696)	0.579
Child-Pugh score, (A vs. B)	2.691 (0.551–2.828)	**0.033**	3.831 (1.164–12.602)	**0.027**
BCLC stage, (0+A vs. B+C)	1.459 (0.434–1.475)	0.356	2.268 (0.793–6.490)	0.127
SHI, (>2.84 vs. ≤2.84)	3.982 (1.806–8.783)	**0.001**	4.417 (1.690–11.542)	**0.002**

*TTR, Time to recurrence; OS, Overall survival; HR, hazard ratio; CI, confidence interval; AFP, α-fetoprotein; BCLC, Barcelona Clinic Liver Cancer; SHI, systemic hepatic-damage index. Bold value represents significant (P < 0.05)*.

### Prognostic value of SHI in the validation cohort

The prognostic value of serum SHI level was further assessed using another independent cohort of 177 HBV-HCC patients. The results were similar to those of the training cohort (Figure 1). HBV-HCC patients with low SHI level have shorter TTR (*p* < 0.001, Figure 1B) and OS (*p* = 0.013, Figure 1B). Cox regression analyses showed that the serum SHI level was an independent indicator of TTR (HR, 3.98; 95% CI, 1.81–8.78; *p* = 0.001; Table 2) and OS (HR, 4.42; 95% CI, 1.69–11.54; *p* = 0.002; Table 2) of HBV-HCC patients. The discrimination ability of SHI was 0.54 (95%CI, 0.45–0.64; Sensitivity, 0.131; Specificity, 0.957 Youden's index, 0.088) and 0.56 (95% CI, 0.45–0.67; Sensitivity, 0.162; Specificity, 0.950; Youden's index, 0.112) for TTR and OS (Figure 1B) respectively, which was higher than other clinical factors.

### Correlation between SHI and clinical characteristics

In the training cohort, there was no significant difference in clinical characteristics, including tumor differentiation and tumor size between the high and low SHI groups. HBV-HCC patients with low SHI were more likely to have vascular invasion (*p* = 0.042, Table 3) in the validation cohort.

**Table 3:** Correlation between the SHI and clinicopathologic characteristics.

**Clinical characteristics**	**Training cohort**				**Validation cohort**		
		**SHI ≤ 2.84 (*N* = 19)**	**SHI > 2.84 (*N* = 169)**	***P***	**SHI ≤ 2.84 (*N* = 13)**	**SHI > 2.84 (*N* = 164)**	***P***
Age	≤ 50	7	62	1.000	7	61	0.250
	>50	12	107		6	103	
Sex	Female	4	22	0.307^a^	1	31	0.468^a^
	Male	15	147		12	133	
AFP, ng/mL	≤ 400	11	128	0.104	7	123	0.110
	>400	8	41		6	41	
ALT, U/L	≤ 75	19	158	0.607^a^	9	90	0.392^a^
	>75	0	11		4	74	
HBsAg	Negative	1	21	0.704^a^	0	21	0.370^a^
	Positive	18	148		13	143	
Liver cirrhosis	No	5	35	0.561	2	41	0.737^a^
	Yes	14	134		11	123	
No. of tumor	Single	18	133	0.130^a^	10	138	0.450^a^
	Multiple	1	36		3	26	
Tumor size, cm	≤ 5	9	110	0.139	6	101	0.378
	>5	10	59		7	63	
Tumor encapsulation	Complete	9	105	0.225	11	110	0.232^a^
	None	10	64		2	54	
Satellite lesion	No	17	148	1.000^a^	12	149	1.000^a^
	Yes	2	21		1	15	
Vascular invasion	No	10	108	0.453	4	100	**0.042**^a^
	Yes	9	61		9	64	
Tumor differentation	I–II	11	105	0.805	7	107	0.548
	III–IV	8	64		6	57	
Child-Pugh score	A	16	161	0.086^a^	11	148	0.625^a^
	B	3	8		2	16	
BCLC stage	0+A	16	121	0.289^a^	8	131	0.156
	B+C	3	48		5	33	

*AFP, α-fetoprotein; ALT, alanine aminotransferase; HBsAg, hepatitis B surface antigen; BCLC, Barcelona Clinic Liver Cancer; SLI, systemic hepatic-damage index*.

a *Fisher exact test. Bold value represents significant (P < 0.05)*.

### The prognostic significance of SHI for HCC patients with negative AFP and early-stage subgroups

**Figure 2:**
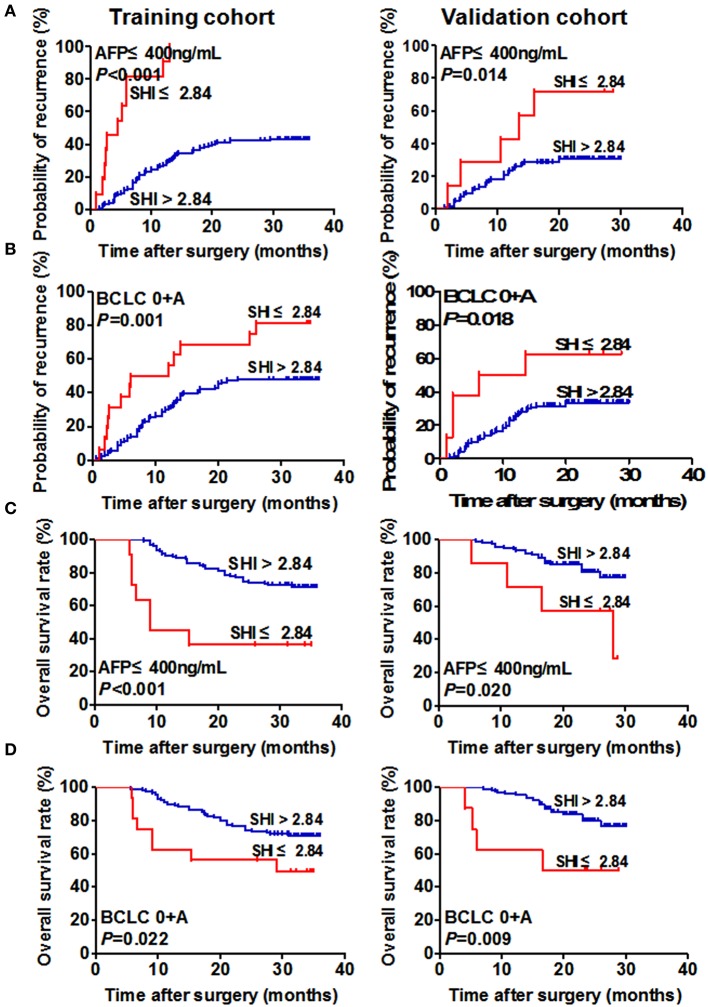
Prognostic significance of SHI of HCC patients in the low-risk and AFP ≤ 400 ng/mL subgroups. Kaplan–Meier analysis of TTR of HCC patients with AFP ≤ 400 ng/mL and BCLC 0+A in the training (**A**, left and **B**, left) and validation (**A**, right and **B**, right) cohorts. Kaplan–Meier analysis of OS of HCC patients with AFP ≤ 400 ng/mL and BCLC 0+A in the training (**C**, left and **D**, left) and validation (**C**, right and **D**, right) cohorts.

The prognostic value of SHI for conventional low-risk or AFP ≤ 400 ng/mL subgroups was further explored. Our results showed that low SHI level was significantly associated with TTR and OS for the variables as follows: AFP ≤ 400 ng/mL for TTR (*p* < 0.001, Figure 2A), BCLC stages 0+A for TTR (*p* = 0.001, Figure 2B), AFP ≤ 400 ng/mL for OS (*p* < 0.001, Figure 2C), BCLC 0+A for OS (*p* = 0.022, Figure 2D) in the training cohort.

In the validation cohort, the prognostic value of SHI for TTR and OS in HBV-HCC patients was maintained in the AFP-negative group (*p* = 0.014 and *p* = 0.020, respectively, Figure 2) and the BCLC 0+A group (*p* = 0.018 and *p* = 0.009, respectively, Figure 2).

### Correlation between SHI and SII and its prognostic significance for HCC patients with detectable SII

**Figure 3:**
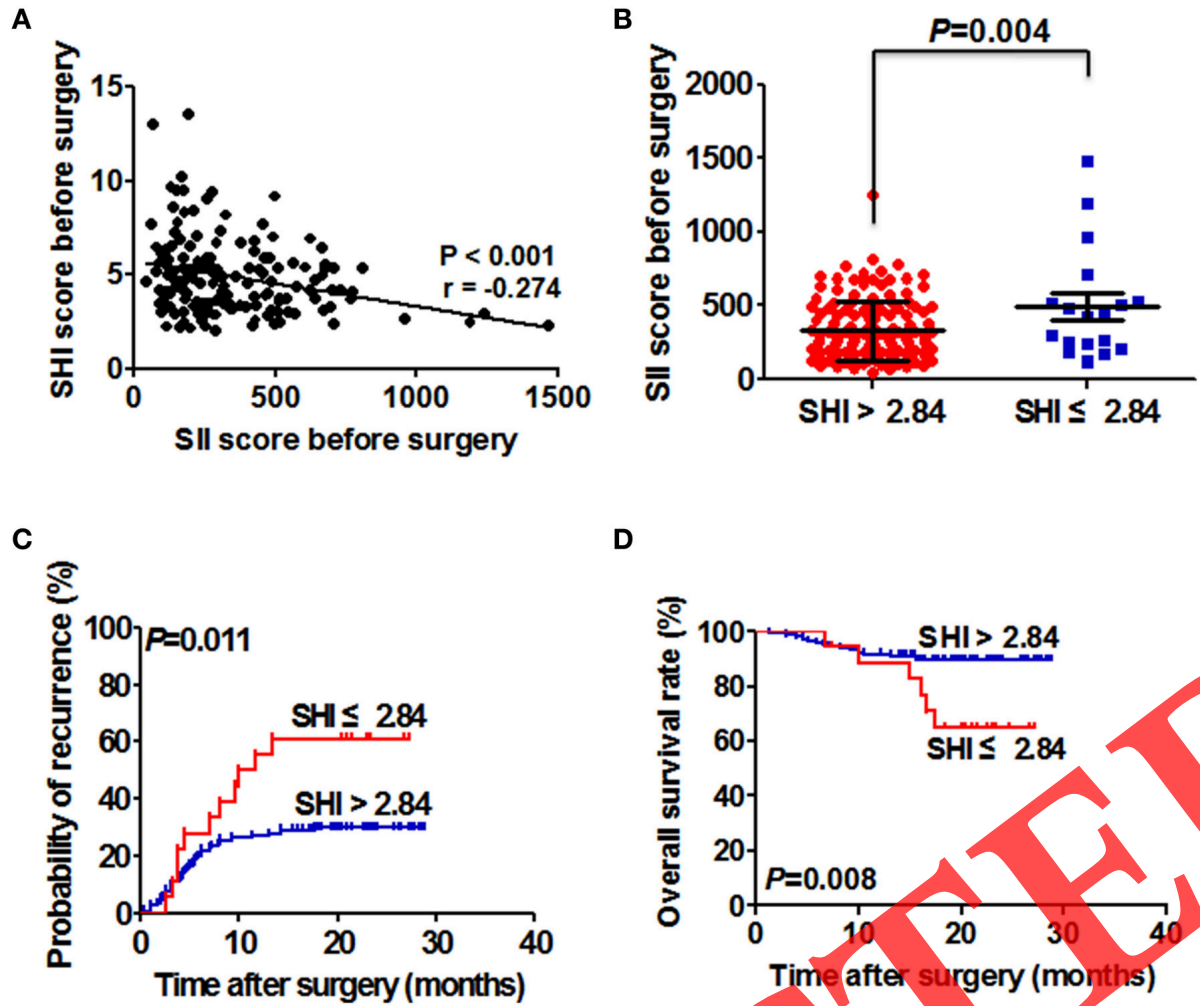
Correlation between SHI and SII, and its prognostic significance in HCC patients with detectable CTC. **(A)** Relationship between SHI and SII. **(B)** The SII score of HCC patients in the low and high SHI groups. **(C)** The Kaplan–Meier analysis of TTR for the SHI in patients with detectable SII. **(D)** The Kaplan–Meier analysis of OS for SHI in patients with detectable SII.

The correlation between perioperative SHI score and perioperative SII score was further explored. The result showed there is a significant negative correlation between SHI and SII (*r* = −0.274; *p* < 0.001; Figure 3A). The SII scores were significantly higher in HBV-HCC patients with low SHI level compared with those with high SHI level (492.31 ± 88.10 vs. 327.30 ± 16.73; *p* = 0.004; Figure 3B).

In light of the close relationship between SHI and SII, we studied the prognostic significance of SHI in HBV-HCC patients with SII. HBV-HCC patients with SHI ≤ 2.84 had higher recurrence rates (61.11 vs. 29.66%; *p* < 0.05) and a shorter TTR (median, 10.9 months vs. not reached; *p* = 0.011) compared with HBV-HCC patients with SHI > 2.84 (Figure 3C). Furthermore, we also found that overall survival time was significantly lower in HBV-HCC patients with SHI ≤ 2.84 than those with SHI > 2.84 (*p* = 0.008, Figure 3D).

## Discussion

The blood lipid level, including triglycerides, TC, HDL, low density lipoprotein, and very low density lipoprotein, were reported to have negative correlation with the severity of liver damage in patients with HBV- or HCV-related chronic liver disease. Moreover, low TC level was reported to be in close association with severe liver fibrosis in these patients. It is implied that liver functional reserve became poorer with the decrease of TC (Vere et al., 2012; Wang et al., 2014). Several studies have shown low serum TC level and low serum HDL level were associated with worse disease-free survival and overall survival in HCC patients undergoing surgical hepatectomy, breast cancer patients, colorectal cancer patients receiving adjuvant chemotherapy, and non-small-cell lung patients with EGFR mutations (Lee et al., 2016; Li et al., 2016; Wang et al., 2016; Zhang et al., 2017). However, these studies focused on the prognostic significance of single index of TC or HDL in post-surgery patients with cancers. Combined with TC and HDL in the prognosis of HCC patient has been not reported.

In the present study, a hepatic-damage–based prognostic score was constructed based on TC and HDL levels, and was an independent factor for predicting recurrence rates and survival time of HCC patients after surgery in the training cohort. Furthermore, we collected another independent cohort of HCC patients to validate the clinical value of SHI levels, and found that the clinicopathalogic characteristics of two cohorts were similar, illustrating the reliability and universality of our finding results. Meanwhile, the measurement of SHI is based on standard laboratory measurements of TC and HDL level, which are routinely performed in the clinical setting. Thus, it is a potential of SHI to be used as a marker for tumor recurrence and treatment responsiveness surveillance, which might provide a powerful test enabling accurate and early decision making to tailor the most effective therapy according to characteristics of individual tumors.

In clinical practice, it is difficult to predict tumor relapse in HCC patients with low recurrence risk. Our study demonstrated that preoperative SHI scores retain their prognostic significance in HCC patients with low recurrence risk whom conventional clinical characteristics offer limited information on predicting tumor recurrence and survival. So far, AFP has been the most extensively useful biomarker for diagnosis and treatment evolution of HCC patients (Sato et al., 1993; Johnson, 2001; Poté et al., 2015). However, it is challenging to monitor tumor recurrence and metastasis in 30–40% of low AFP levels patients with HCC (Johnson, 2001; Forner et al., 2012). Here, we have also shown that detection of the preoperative SHI level is a convenient and good tool for predicting recurrence in low AFP levels patients with HCC. The prognosis predictive value of SHI level in low recurrence risk subgroups would be that clinicians could easily identify HCC patients at high risk of tumor recurrence and metastasis, and provide targeted rational adjuvant therapy after surgery. To date, detection of SHI level has been served as a routine clinical liver function test with commercially available kits. Determination of SHI can be standardized easily to provide accuracy and reproducibility, strengthening its practical significance for determining the most effective therapy for each HCC patient at an early stage.

Recent evidence suggests that chronic immuno-inflammatory cells involve in tumorigenesis and tumor progression (Baecklund et al., 2006; Smedby et al., 2006; Lim et al., 2007). Reducing serum HDL concentration leads to chronic inflammation because of its anti-inflammatory properties. Thus, low HDL level may predict the changes of systemic inflammation and the risk of inflammation-induced tumor. Furthermore, high HDL may protect against tumor, which modulates inflammatory responses with suppressing chemotactic activity of lymphocytes and monocytes and inhibiting cytokine-induced expression of endothelial cell adhesion molecules, and protecting the integrity of lymphocytes from oxidative damage (Cockerill et al., 2001; Nofer et al., 2002; Tietge et al., 2003; Papageorgiou et al., 2016). All of those might suggest SHI affect tumor progression through modulating inflammatory responses. When analyzing only the patients with detectable SII, patients with low SHI level had a higher recurrence rate and a shorter TTR than patients with high SHI level. This suggests that SHI are unable to influence tumor cells without assistance from immune and inflammatory cells. In addition, we also found that decreased SHI was associated with vascular invasion, indicating a more aggressive phenotype. Release of tumor cells into circulation is a multiple-step procedure, which includes a great number of ingredients, and SHI acts as one of the major molecules in this procedure (Hu et al., 2014). Hence, based on our data, we believe that SHI is a significant negative regulator of SII (*p* < 0.001), though the correlation was relatively weak (*r* = −0.274). Taken together, these findings indicated that the SHI might increase the SII level by preventing tumor formation, affecting tumor cell survival in the circulation, leading to higher recurrence rates and poor outcomes in HCC patients with low serum SHI levels. Thus, improving the SHI level in HCC patients might be a promising therapeutic strategy to limit tumor recurrence and metastasis in HCC patients after surgery.

Our data shows that SHI can independently predict TTR and OS for HCC patients with resection. Furthermore, continuous monitoring of SHI in peripheral blood of HCC patients might provide available information for managing and facilitating treatment of HCC patients. The role of SHI in increasing SII scores can probably serve as the reason of high recurrence rate and poor prognosis of HCC patients with low SHI level. The cheap and easy detection of SHI level makes SHI serve as a convenient biomarker for predicting prognosis value of HCC individual in future clinical practice. Moreover, improving SHI level in peripheral blood of HCC patients might be a promising therapeutic strategy to reduce the possibility of recurrence and metastasis of HCC patients with resection. However, our study still exist several limitation. In the study, some recognized factors, such as tumor number, tumor size, and tumor differentiation are not independent risk factors for TTR and OS for HCC patients. It might be the reason of short follow-up time and small size of the cohort relatively. Moreover, it should also be noted that there is a hepatitis B virus-positive background in most HCC patients in China. Therefore, a large-cohort, multicenter, long-term study including HCC patients from different background and country is needed to validate the prognostic value of SHI, and this is currently conducted in our center. Furthermore, a comprehensive study of the mechanisms of between SHI and HCC cells also needs to be explored.

## Author contributions

XG and FW conceived and designed the experiments. XG and SZ performed the research, conducted the data analyses, and wrote the manuscript. HC, YW, and CY contributed to the clinical data collection. FW revised the manuscript and coordinated the research team. All authors have read and approved the final manuscript.

### Conflict of interest statement

The authors declare that the research was conducted in the absence of any commercial or financial relationships that could be construed as a potential conflict of interest.

## Supplementary material

Supplementary Table 1 The area under curve for TTR and OS in the training and validation cohort. TTR, Time to recurrence; OS, Overall survival; AUC, the area under curve; CI, confidence interval; AFP, α-fetoprotein; BCLC, Barcelona Clinic Liver Cancer; SHI, systemic hepatic-damage index.

Supplementary Figure 1 The optimal cutoff value of SHI was selected by X-tile 3.6.1 software. SHI, systemic hepatic-damage index.

## Abbreviations

HCC: Hepatocellular carcinoma
TTR: time to recurrence
OS: Overall survival
AFP: α-fetoprotein
BCLC: Barcelona Clinic Liver Cancer
SHI: systemic hepatic-damage index
SII: systemic immune-inflammation index.
