# Supplementary material for: Systemic Hepatic-Damage Index for Predicting the Prognosis of Hepatocellular Carcinoma after Curative Resection
Source: Front Physiol. 2017 Jul 18;8:480. doi: 10.3389/fphys.2017.00480 (PMC5513961; doi:10.3389/fphys.2017.00480)
Supplement: Supplementary Table 1 — The area under curve for TTR and OS in the training and validation cohort. TTR, Time to recurrence; OS, Overall survival; AUC, the area under curve; CI, confidence interval; AFP, α-fetoprotein; BCLC, Barcelona Clinic Liver Cancer; SHI, systemic hepatic-damage index. [file Table1.docx]

| **Supplemental Table 1.**  **The Area Under Curve for TTR and OS in the training and validation cohort** | | | | |
| --- | --- | --- | --- | --- |
| **Variables** | **TTR** | | **OS** | |
|  | AUC | 95% CI | AUC | 95% CI |
| **Training cohort** |  |  |  |  |
| AFP, ng/ml (>400 vs.≤400) | 0.502 | 0.415-0.581 | 0.569 | 0.480-0.658 |
| No. of tumors, (multiple vs. single) | 0.507 | 0.424-0.590 | 0.512 | 0.394-0.581 |
| Tumor size, cm (>5 vs. ≤5) | 0.502 | 0.419-0.585 | 0.516 | 0.422-0.609 |
| Tumor encapsulation, (none vs. complete) | 0.513 | 0.430-0.596 | 0.516 | 0.423-0.609 |
| Vascular invasion, (yes vs. no) | 0.525 | 0.442-0.608 | 0.528 | 0.436-0.621 |
| Tumor differentiation, (III-IV vs. I-II) | 0.513 | 0.430-0.596 | 0.536 | 0.442-0.629 |
| BCLC stage, ( 0+A vs. B+C) | 0.564 | 0.481-0.647 | 0.580 | 0.491-0.668 |
| SHI, (>2.84 vs. ≤2.84) | 0.567 | 0.485-0.649 | 0.538 | 0.443-0.633 |
| **Validation cohort** |  |  |  |  |
| AFP, ng/ml (>400 vs. ≤400) | 0.560 | 0.470-0.651 | 0.537 | 0.431-0.644 |
| No. of tumors, (multiple vs. single) | 0.513 | 0.422-0.603 | 0.533 | 0.426-0.640 |
| Tumor size, cm (>5 vs. ≤5) | 0.536 | 0.446-0.626 | 0.523 | 0.418-0.629 |
| Tumor encapsulation, (none vs. complete) | 0.546 | 0.456-0.637 | 0.556 | 0.450-0.663 |
| Vascular invasion, (yes vs. no) | 0.536 | 0.446-0.625 | 0.522 | 0.417-0.626 |
| Tumor differentiation, (III-IV vs. I-II) | 0.516 | 0.426-0.606 | 0.520 | 0.416-0.624 |
| BCLC stage, ( 0+A vs. B+C) | 0.511 | 0.421-0.601 | 0.535 | 0.428-0.642 |
| SHI, (>2.84 vs. ≤2.84) | 0.562 | 0.481-0.655 | 0.556 | 0.447-0.666 |
| Abbreviations: TTR, time to recurrence; OS, Overall survival; AUC, the area under curve; CI, confidence interval; AFP, α-fetoprotein; BCLC, Barcelona Clinic Liver Cancer; SHI, systemic hepatic-damage index. | | | | |
