# Supplementary figures and images for: Systemic Hepatic-Damage Index for Predicting the Prognosis of Hepatocellular Carcinoma after Curative Resection
Source: Front Physiol. 2017 Jul 18;8:480. doi: 10.3389/fphys.2017.00480 (PMC5513961; doi:10.3389/fphys.2017.00480)

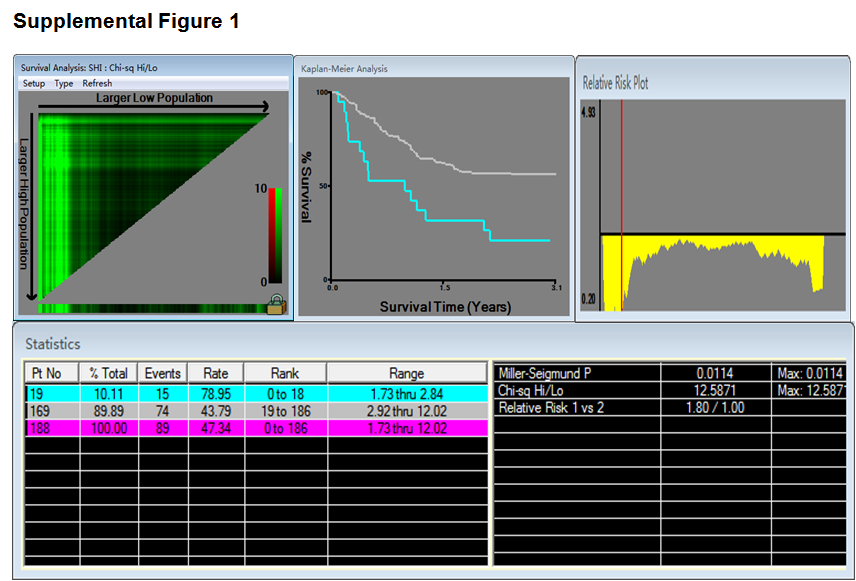

Supplement: Supplementary Figure 1 — The optimal cutoff value of SHI was selected by X-tile 3.6.1 software. SHI, systemic hepatic-damage index. [file Image1.tif]
